# The P2X7 Receptor: A Key Player in Immune-Mediated Bone Loss?

**DOI:** 10.1155/2014/954530

**Published:** 2014-01-16

**Authors:** Torben Madsen Kvist, Peter Schwarz, Niklas Rye Jørgensen

**Affiliations:** ^1^Research Center for Ageing and Osteoporosis, Departments of Medicine and Diagnostics, Glostrup Hospital, 2600 Glostrup, Denmark; ^2^Faculty of Health Sciences, Copenhagen University, 2100 Copenhagen Ø, Denmark

## Abstract

Inflammatory diseases are often multiorganic diseases with manifestations not related directly to the primary affected organ. They are often complicated by a generalized bone loss that subsequently leads to osteoporosis and bone fractures. The exact mechanism for the accompanying bone loss is not understood in full detail, but factors such as glucocorticoid treatment, immobilization, malnutrition, and insufficient intake of vitamin D play a role. However, it has become evident that the inflammatory process itself is involved and the resulting bone loss is termed immune-mediated bone loss. It stems from an increase in bone resorption and the pro-inflammatory cytokines tumor necrosis factor alpha and interleukin 1 beta and has been shown to not only mediate the inflammatory response but also to strongly stimulate bone degradation. The purinergic P2X7 receptor is central in the processing of these two cytokines and in the initiation of the inflammatory response, and it is a key molecule in the regulation of both bone formation and bone resorption. The aim of this review is therefore to provide evidence-based novel hypotheses of the role of ATP-mediated purinergic signalling via the P2X7 receptor in immune-mediated bone loss and -osteoporosis.

## 1. Bone Loss in Chronic Inflammatory Diseases

Inflammatory diseases are frequent in the western world. It is estimated that the prevalence of autoimmune diseases is 3% and that the life time risk of a rheumatic autoimmune inflammatory disease is about 5% for males and 8.3% for females [1]. Inflammatory diseases are often multiorganic diseases with manifestations not related directly to the primary affected organ, including a generalized bone loss leading to osteoporosis. Osteoporosis is characterized by low bone mass with microarchitectural changes in bone, which leads to an increased susceptibility to fractures. Hip fractures lead to significant morbidity, such as severe pain, disabilities, decreased mobility, impaired respiratory function, and increased mortality. Systemic osteopenia is frequently observed [2, 3]. The number of patients with accompanying bone loss depends on the inflammatory disease. In patients with recently diagnosed rheumatoid arthritis more than 10% are osteoporotic, while approximately 25% are osteopenic [4–6]. In patients with inflammatory bowel disease (IBD), the risk of secondary bone loss is reported as high, that is, 51–77% suffering osteopenia and 17–28% suffering from osteoporosis [7]. In IBD, the fracture risk may be increased by about 40% [8]. Also in patients with chronic obstructive pulmonary disease (COPD), 68% either had osteopenia or were suffering from osteoporosis [9].

The clinical pathogenesis of bone loss in chronic inflammatory diseases is multifactorial, where especially the use of glucocorticoids for the treatment of the inflammatorys states has been shown to have deleterious effects on the skeleton [10]. However, also other factors such as malnutrition, low vitamin D levels, immobility or inactivity, and changes in the endocrine system might all partly be responsible. Finally, the inflammatory process itself is proposed as having an effect on the skeleton leading to alterations in bone metabolism and subsequent bone loss. However, to what extent the disease itself might cause bone loss is disputable.

The pathophysiological mechanisms of inflammation-induced bone loss are complex and are thought to be mediated through effects on both bone resorption and bone formation [10], as the effects are the increased osteoclastic bone resorption and inhibition of osteoblastic bone formation [6]. Some of our knowledge on the signalling pathways involved in the changes in bone remodelling arises from the first model of generalized osteoporosis resulting from chronic inflammation developed by K. J. Armour and K. E. Armour [11]. At the molecular level, especially increased levels of receptor activator of nuclear factor kappa ligand (RANKL), interleukin 1 (IL-1), interleukin 6 (IL-6), tumor necrosis factor alpha (TNF-*α*), and prostaglandin E2 (PGE-2) are responsible for the induction of bone loss [11]. Furthermore, INOS seem to be involved as studies an iNOS knockout mice were shown to have higher trabecular bone volume and a lower rate of osteoblast apoptosis [12, 13]. TNF-*α* and IL-1*β* are known as potent inflammatory signals and also as regulators of osteoclast formation and activity [14–16]. Increased levels of TNF-*α* and IL-1*β* are seen after estrogen withdrawal in relation to menopause in women and might, at least partially, be responsible for the rapid perimenopausal bone loss. In contrast, TNF-*α* inhibitors have been shown to reduce both joint destruction in rheumatoid arthritis and the associated bone loss [17].

In the middle of the above mentioned signalling pathways stands the purinergic P2X7 receptor, which has also been demonstrated to play a pivotal role in the regulation of both bone formation and bone resorption. The P2X7 receptor could therefore prove to be a key mediator of inflammatory-induced bone loss. Thus, the aim of this review is to review the current state of evidence for the role of the P2X7 receptor in inflammatory-induced bone loss and to discuss the P2X7 receptor as a possible pharmacological target for inhibiting bone loss or even in the treatment of osteoporosis in patients with chronic inflammatory diseases.

## 2. P2X7 Receptors and ATP-Mediated Purinergic Signaling in the Immune System during Inflammation

ATP is a widely used extracellular signalling molecule. It is believed that small transient increases serve as basic physiological signalling and higher levels are associated with cell death and serve as a key danger signal [18, 19]. ATP is present in high amounts in intracellular stores, but under normal physiological conditions only in very low extracellular levels. ATP is quickly degraded enzymatically after release, and as it is the natural ligand of most P2 purinergic receptors, ATP and its breakdown products activate a wide range of P2 receptors. Low levels of ATP lead to suppressed inflammation and immune deviation [20], while high levels of ATP are associated with tissue stress and damage, as ATP is released by high cell turnover and necrosis by almost all tissues. High levels of extracellular ATP are present at sites with infection, inflammation, and conditions with high cell turnover as seen in cancer [21], and in bone tissue high ATP levels may occur after tissue and cell injury such as microcracks and even after fractures. Using a luciferase-expressing transgenic mouse, it has been shown that high levels of extracellular ATP are released during inflammation and after intraperitoneal injection of cancer cells [22, 23]. As high extracellular ATP concentrations are required for the activation of the P2X7 receptor in both bone and the immune system and as high ATP levels are predominantly released during inflammatory conditions and other conditions of tissue injury, it is highly likely that the effects of the released ATP during these conditions are mediated through the P2X7 receptor.

P2X7 receptors are highly expressed in cells of the immune system (Table 1) with the highest expression seen in macrophages followed by dendritic cells, monocytes, natural killer cells, B-lymphocytes, T-lymphocytes, and erythrocytes in descending order [63–65]. The P2X7 receptor is part of the multiprotein complex called the inflammasome [66], which is central in the events during initiation of the inflammatory response in the innate immune system [34], and especially in the initiation phase macrophages, monocytes and dendritic cells are predominantly involved in the innate immune response. Here, P2X7 receptor activation induces activation of the NALP3 inflammasome leading to conversion of procaspase-1 to caspase-1 [67, 68]. Caspase-1 is involved in processing and release of IL-1 and IL-18 with subsequent systemic effects in the immune system and possibly also in bone. Also, caspase-1 activation can lead to apoptosis of the activated cell. Release mechanisms are not fully known [69], but opening of Pannexin-1 channels has been proposed as being implicated in IL-1*β* release [70]. P2X7 is also involved in the release of other cytokines, including TNF-*α* [34, 71], and lower levels of TNF-*α* release are linked to polymorphisms of P2X7 alleles with reduced pore formation [72].

## 3. P2X7 Receptors in Regulation of Bone Metabolism

Bone is a highly specialized tissue with a high metabolic activity. It consists of organic collagen matrix with mineral deposits in the form of hydroxyapatite and three distinct cells types; the osteoblasts are the bone forming cells and the osteoclasts are the bone degrading cells. Both the osteoblasts and the osteoclasts primarily reside on the bone surface, while the third cell type, the osteocyte, is embedded inside the mineralized bone matrix, where they act as mechanosensors of the bone, sensing and transducing mechanical signals into biological signals of bone turnover. Bone remodelling is highly organized taking place at millions of sites being regulated by systemic and local factors, autocrine/paracrine signals, and mechanical stimuli [73]. Bone cells release nucleotides into the extracellular environment to provide highly localized and transient signals that regulate bone formation and bone resorption [48]. Thus, ATP acts as an important, local signalling mechanism, but due to rapid extracellular degradation by enzymes, the range of signal is presumably limited. It mediates direct cell-to-cell communication [74–79]. The sources of nucleotides/ATP in bone are multiple; osteoblasts [52] and osteocytes [80] release ATP in response to mechanical stimulation. Nucleotides can be released by cells in the bone marrow (immune cells and hematopoietic cells), and theoretically, ATP may also be released from osteocytes as a result of cell damage after microcracks in the bone tissue. Thus, ATP might very well be one of the most important extracellular regulatory molecules in the skeleton [81, 82].

**Figure 1:**
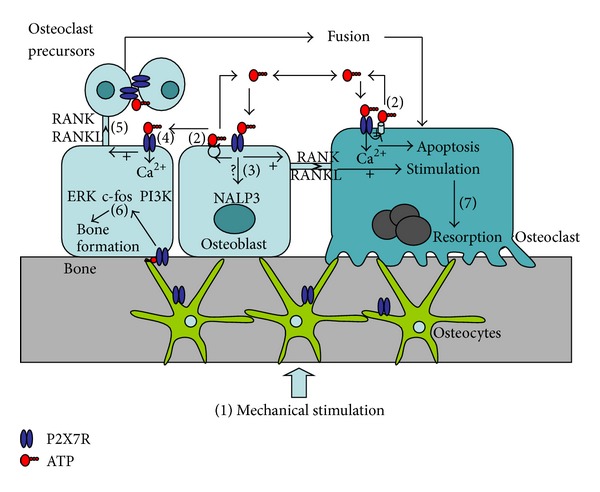
The physiological functions of the P2X7 receptor in bone cells. Numbers in brackets refer to published cellular functions associated with P2X7 receptor activation and/or expression. (1) Mechanostimulation induces ATP release. (2) Modulation of ATP release by the P2X7 receptor. ATP release from osteoblasts involves vesicular exocytosis. In osteoclasts, ATP release is associated with P2X7 activation, but the exact mechanism has not yet been determined. (3) Osteoblasts may be a source of local cytokine release possibly through the P2X7 inflammasome pathway. (4) ATP is released from osteoblasts upon mechanical and nucleotide stimulation and mediates paracrine signalling to neighboring cells via P2X7 receptor activation. (5) P2X7 receptor activation induces RANKL expression in osteoblasts and subsequently formation of osteoclasts from mononuclear precursors. (6) P2X7 receptors activation is coupled to intracellular signalling pathways in osteoblasts that induce cell growth and bone formation. (7) In osteoclasts, P2X7 receptor activation is linked to fusion of mononuclear osteoclast precursors and to apoptosis and cell survival hand to activation of osteoclastic bone resorption.

Among all the P2 receptors, the P2X7 receptor is the most widely studied in relation to bone (Table 2). The most important functions of the P2X7 receptor in bone are shown in Figure 1. Its expression in osteoblasts is a differentiation-dependent expression and the receptor is mainly expressed in mature bone forming osteoblasts [48]. High concentrations of ATP are needed to activate the P2X7 receptor and it has been controversial whether the receptor has any physiological functions in osteoblasts. In vitro concentrations of ATP above 1 mM partly inhibit bone formation especially mineralization [83]. This is a high concentration of ATP, and it may only occur in vivo as a result of cell damage including microcracks in the bone tissue. Consequently, the effects are thought to be caused mainly by P2X7 and, like in the immune system, activation of P2X7 receptors by ATP in the skeleton may be a danger signal of tissue or cell injury. In contrast, constitutive ATP release in osteoblasts has been shown to be at low levels, 0.5–1 nmol/mL under normal conditions [84]. The breakdown of ATP by ectonucleotidases results in high levels of PPi, which is also know to inhibit bone mineralization. PPi could account for some of the observed effects of ATP on bone cells. P2X7 receptor activation in osteoblasts results in a number of cellular events including activation of apoptosis [85], fluid shear stress-induced ERK1/2 activation [86] and nuclear factor kappaB (NF*κ*B) translocation [84], membrane blebbing [87], and induction of receptor activator of nuclear factor kappa-B ligand (RANKL) [54], which is important in stimulating fusion of mononuclear osteoclast precursors and activating osteoclastic bone resorption. However, the full functional role of P2X7 receptors in osteoblasts is not yet fully elucidated. Also osteocytes express P2X7 receptors [88], but the function is virtually unknown. However, it has been shown that P2X7 receptors are important for a normal anabolic response to physical stimulation of the skeleton [89], so it could be speculated that P2X7 receptors are involved in the mechanotransductory cascade in osteocytes as osteocytes release large amounts of ATP upon mechanical stimulation [80, 90].

Osteoclasts are derived from the same monocytic precursors as the macrophages, and have many similarities with these. The osteoclast is also the bone cell where P2X7 receptor expression has been investigated most extensively. P2X7 receptors are expressed at all stages of differentiation with the highest expression on the mature osteoclast, where expression is four to five times higher than in earlier stages of the osteoclast [91], but relatively high levels are found on all cells in the osteoclasts lineage. The P2X7 receptor activation couples to a number of intracellular signalling pathways in osteoclasts including NF*κ*B, which is a transcription factor essential for osteoclast development [92] and osteoblast function [93]. It is also involved in cell proliferation, apoptosis, and inflammation [73, 94, 95]. P2X7 receptors also activate nuclear factor of activated T-cells (NFAT) [96] which is also coupled to cell proliferation and growth. Also other pathways are activated through the P2X7 receptor such as phosphoinositide 3-kinase (PI3 K/AKT/mTOR) [43] and rho-associated protein kinase (ROCK), suggesting a role for the receptor in regulation of osteoclast apoptosis and cell motility. In line with the signalling pathways activated, the receptor is involved in formation of multinucleated osteoclasts [61] partially through control of the fusion of osteoclast precursor cell membranes osteoclasts and blockade of this receptor inhibits resorption [82]. However, using mouse P2X7 knockout models, it has been shown that, though P2X7 receptors are involved in formation of multinucleated osteoclasts, they are not crucial for the fusion of osteoclast precursors as osteoclasts still form in the knockout animals [97]. In addition to the effects on osteoclasts proliferation and growth, P2X7 receptor activation has effects on bone resorption through secretion of bone degrading enzymes such as matrix metalloproteases [98] and cathepsin [99].

One of the possible links between the immune system and regulation of bone metabolism came from the P2X7 receptor knockout models where macrophages from the P2X7 receptor null mice showed reduced interleukin 1 (IL-1) production [100]. Moreover, in an arthritis model, based on injecting anticollagen antibodies into the animals, an attenuated inflammatory response was found in the P2X7 receptor null animals compared to their wild type littermates [39, 100]. Unfortunately, the findings from the in vivo studies of the P2X7 knockout models on effects in bone are somewhat conflicting, primarily caused by the fact that neither of the two available knockout models are true knockouts as some splice variants of the P2X7 receptor are still expressed in some tissues [101–104]. In the study by Ke et al. (using the “Pfizer” knockout), the P2X7 null animals displayed a bone phenotype comparable to misuse [97]. Moreover, reduced sensitivity to mechanotransduction was found [89]. In contrast, in another P2X7 knockout model (using the “GSK” knockout), Gartland and colleagues did not find any obvious skeletal phenotype [85]. Later, Nicke et al. demonstrated that after backcrossing the “GSK” knockout into the BALB/cJ background as the original C6 background already carried a naturally occurring P451L mutation in the P2X7 receptor [102], P2X7 null animals had higher bone mass than their wildtype littermates [105–107]. Though in vivo studies of P2X7 receptor function are conflicting, they demonstrate that the P2X7 receptor is important in bone metabolism, and its exact role cannot be depicted precisely from these studies.

In support of the role of the P2X7 receptor in regulating bone turnover in humans and in the pathogenesis of osteoporosis, recent studies have demonstrated that a number of P2X7 receptor single nucleotide polymorphisms are associated with bone loss and vertebral fracture in postmenopausal women [108–112]. Generally, loss of function of the receptor seems to be associated with increased fracture risk and high rate of bone loss after menopause, while gain of function is associated with reduced fracture risk and low rate of bone loss.

## 4. P2X7 Receptors as the Link between the Immune System and Bone in Chronic Inflammatory Diseases

**Figure 2:**
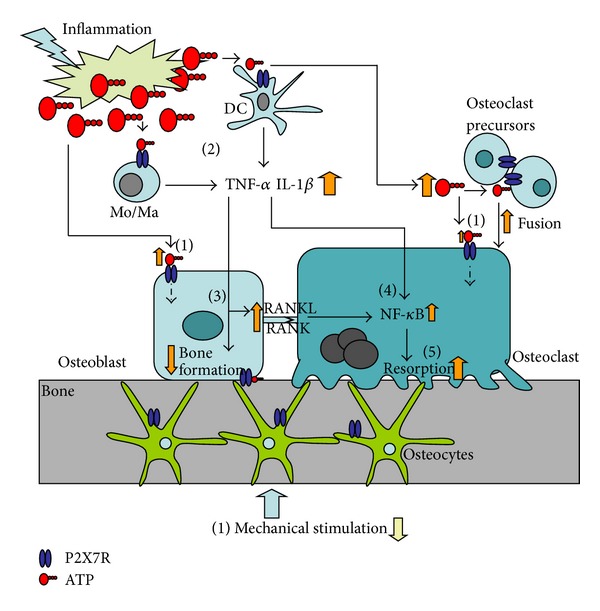
The theoretical involvement of the P2X7 receptor in inflammatory-induced bone loss. The inflammatory process involves release of large amounts of ATP that activates P2X7 receptors on immune cells and possibly also P2X7 receptors on bone cells. (1) P2X7 receptors on bone cells are activated by ATP released from tissue/cell injury at the site of inflammation. (2) P2X7 receptors on monocytes (Mo), macrophages (Ma), and dendritic cells (DC) at the site of inflammation are activated by ATP released from cell/tissue injury. P2X7 receptor activation on these cells induces K^+^ efflux and activation of the NALP3 inflammasome leading to processing and release of proinflammatory cytokines such as interleukin-1*β* (IL-1*β*) and tumor necrosis factor-*α* (TNF-*α*). (3) TNF-*α* inhibits osteoblastic function (reduces bone formation and cell growth). (4) Both IL-1*β* and TNF-*α* converge to nuclear factor *κ*B (NF-*κ*B) and subsequently stimulating osteoclastic bone resorption. (5) TNF-*α* is directly proresorptive. In addition (not shown on figure), expression of receptor activator of nuclear factor ligand (RANKL) on inflammatory cells is increased and upon binding to receptor activator of nuclear factor (RANK) on mononuclear osteoclast precursors stimulating formation of multinucleated osteoclasts and activation of osteoclastic bone resorption.

As evidenced above, it is obvious that the P2X7 receptor is central to both the innate inflammatory response and to the regulation of bone turnover, and there seems to be effects that are differentiated depending on low levels of ATP and high levels of ATP. A number of the P2X7-coupled pathways are important in both the immune response and in the control of bone resorption, as bone resorption is increased significantly in inflammatory-induced bone loss. During the inflammatory process the large amounts of ATP are released; ATP is a natural ligand for most P2 receptors and an inflammation-induced ATP release would therefore activate a range of P2 receptors, including the P2X7 receptor which requires high concentrations of ATP to be activated. As ATP is rapidly degraded extracellularly, systemically increased levels of ATP are unlikely. Skeletal effects of ATP-mediated purinergic signalling through P2X7 receptors may therefore occur via three mechanisms. The first is the general skeletal bone loss, which might be induced through local activation of P2X7 receptors on immune cells inducing IL-1*β* and TNF-*α* production, processing and release to the circulation where it induces a generalized increase in bone resorption through activation of osteoclasts in all parts of the skeleton (Figure 2). Secondly, ATP release as part of the inflammatory process could activate bone cells locally as the immune system and bone are in close contact both in the bone marrow and in affected joints. Here, activation of P2X7 receptors in bone cells could activate osteoclasts both through a direct activation of osteoclasts precursors to form mature multinucleated bone-resorbing osteoclasts and through an indirect activation via stimulation of osteoblastic P2X7 receptors and upregulation of RANKL on osteoblasts, thereby inducing osteoclasts formation and activity (Figure 2). Finally, due to reduced mobility of patients due to their primary inflammatory diseases, a reduced activation of P2X7 receptors in osteoblasts, usually mediating mechanotransduction and anabolic effects, results in reduced bone formation, which further aggravates the effects of the disease on the skeleton.

The question then arises whether P2X7 receptor polymorphisms affect the risk of inflammatory-induced bone loss. One study has examined the association between P2X7 receptor polymorphisms and the risk of rheumatoid arthritis or systemic lupus erythematosus; no association could be demonstrated [113]. However, no studies have so far examined the association between chronic inflammatory diseases and the risk or severity of bone loss, so this is purely speculative. However, if P2X7 receptors are involved in an inappropriate activation of the immune system in autoimmune diseases, loss-of-function polymorphisms might protect against these diseases and the associated bone loss. This is naturally in contrast to what has been shown in normal, healthy subjects as described above, but in states of chronic inflammation there is a pathological activation of the immune system, an extraordinary high release of ATP, and thereby a pathological activation of bone turnover. In this case, a reduction in P2X7 receptor function might very well protect against the increased bone resorption seen in chronic inflammatory diseases. However, this still needs to be elucidated.

## 5. P2X7 Receptors as Targets for Pharmacological Treatment of Inflammatory Diseases

The P2X7 receptor has been suggested as a promising therapeutic target in relation to inflammatory diseases [114–116] Recently, a number of clinical trials using a P2X7 receptor inhibitor have been reported [117–120]. These studies have generally shown disappointing results in terms of overall disease control; results from AstraZeneca on the P2X7 antagonist AZD9056 in Phase IIa were promising for treatment of rheumatoid arthritis, but in the six month phase IIb study, no clinically meaningful effect of the compound in the treatment of rheumatoid arthritis could be demonstrated [121]. Another P2X7 receptor inhibitor developed by Pfizer, Ce-224,535, was tested in a 12-week phase II trial in patients with rheumatoid arthritis insufficiently controlled by methotrexate. The results from this study were equally disappointing in terms of controlling the overall disease activity and were also disappointing [122] indicating that P2X7 receptor inhibition is not the way to go on in terms of disease control in rheumatoid arthritis. This could be due to the complex nature of the pathogenesis of the diseases and the complex nature of the inflammation. However, in the preclinical rat studies with AZD9056 encouraging results on the bone component of the disease could be demonstrated [123] Effects on radiographic progression and especially on erosions could be demonstrated; radiology and histology showed a dose-dependent reduction in bone lesion scores and bone resorption, synovial inflammation and pannus formation, and finally a reduced incidence of osteoclastic giant cells [123]. In conclusion, P2X7 antagonists do not seem to affect the overall acute phase part of rheumatoid arthritis. However, the findings on the bone compartment in both the animal preclinical and the clinical phase II study are nevertheless encouraging, and P2X7 receptor inhibition could very well turn out to have important effects of inhibiting bone resorption associated with chronic inflammatory diseases and thereby reducing the skeletal unwanted effects of these debilitating diseases. However, longer-term studies designed to document these effects are warranted.

## 6. Summary

In summary, the P2X7 receptor is a key molecule in the activation of the innate immune response. It is also central in the regulation of bone turnover, and ATP-mediated purinergic signalling might very well be the key to understand inflammatory-induced bone loss. Though P2X7 receptor inhibition has failed to control the short-term clinical symptoms of rheumatoid arthritis, preliminary results from preclinical and clinical testing indicate that it may prove to be powerful in reducing the unwanted skeletal effects of acute inflammatory states. However, more studies are warranted to demonstrate the clinical utility of the P2X7 receptor inhibitor in these indications.

## Figures and Tables

**Table 1:** Effects of P2X7 receptors in immune cells.

Cell type	Cellular effect	Reference
Monocyte/macrophage	Activation of the inflammasome	[24, 25]
	Secretion of cytokines: IL1b, IL18, and TNF-*α*	[26–28]
	Modulation of phagocytosis	[29]
	Macrophage death	[30]
	Promotion of multinucleated macrophages	[31, 32]
	Cathepsin release	[33]
	Enhance NO production and NOS2 expression	[34]

Dendritic cells	Maturation	[35]
	Apoptosis	[36, 37]
	Inflammasome activation	[38]
	Secretion of proinflammatory cytokines (IL1b, IL18, and TNF-*α*).	[38]
Lymphocytes	Apoptosis	[35, 39]
	Proliferation	[40, 41]

**Table 2:** Published effects of P2X7 receptors in immune cells.

Cell type	Cellular effect	Reference
Osteoblast	RANKL expression (high ATP levels)	[42]
	Cell growth and bone formation though c-fos, ERK, PI3K, and COX	[43–47]
	Apoptosis	[48]
	Bone formation, membrane blebbing	[47, 49]
	Mineralization of bone matrix	[50]
	Mechanotransduction/mediate anabolic response to mechanical stimulation of bone	[51]
	Mediate ERK1/2 via fluid shear stress	[44]
	ATP release	[42, 52, 53]
	Fluid shear stress-induced NF-*κ*B translocation	[54]
	Processing and secretion of cytokines	[55]

Osteoclast	Apoptosis/survival	[56, 57]
	Intercellular calcium signalling	[58]
	ATP release	[59]
	Precursor cell fusion and osteoclast cell fusion	[57, 60]
	Activation of NF*κβ*	[61, 62]

## References

[1] Crowson CS, Matteson EL, Myasoedova E (2011). The lifetime risk of adult-onset rheumatoid arthritis and other inflammatory autoimmune rheumatic diseases. *Arthritis and Rheumatism*.

[2] Goldring SR, Gravallese EM (2000). Mechanisms of bone loss in inflammatory arthritis: diagnosis and therapeutic implications. *Arthritis Research*.

[3] Goldring SR, Schett G, Lorenzo J, Choi Y, Horowitz M, Takayanagi H (2011). The role of the immune system in the bone loss of inflammatory arthritis. *Osteoimmunology*.

[4] Güler-Yüksel M, Bijsterbosch J, Goekoop-Ruiterman YPM (2007). Bone mineral density in patients with recently diagnosed, active rheumatoid arthritis. *Annals of the Rheumatic Diseases*.

[5] Mundy GR (2007). Osteoporosis and inflammation. *Nutrition reviews*.

[6] Schett G, David JP (2010). The multiple faces of autoimmune-mediated bone loss. *Nature Reviews Endocrinology*.

[7] Andreassen H, Rungby J, Dahlerup JF, Mosekilde L (1997). Inflammatory bowel disease and osteoporosis. *Scandinavian Journal of Gastroenterology*.

[8] Bernstein CN, Leslie WD (2004). Review article: osteoporosis and inflammatory bowel disease. *Alimentary Pharmacology and Therapeutics*.

[9] Jørgensen NR, Schwarz P, Holme I, Henriksen BM, Petersen LJ, Backer V (2007). The prevalence of osteoporosis in patients with chronic obstructive pulmonary disease—a cross sectional study. *Respiratory Medicine*.

[10] Hardy R, Cooper MS (2009). Bone loss in inflammatory disorders. *Journal of Endocrinology*.

[11] Armour KJ, Armour KE (2003). Inflammation-induced osteoporosis. The IMO model. *Methods in Molecular Medicine*.

[12] Armour KJ, Armour KE, van’t Hof RJ (2001). Activation of the inducible nitric oxide synthase pathway contributes to inflammation-induced osteoporosis by suppressing bone formation and causing osteoblast apoptosis. *Arthritis *S* Rheumatism*.

[13] Armour KE, Van ’T Hof RJ, Grabowski PS, Reid DM, Ralston SH (1999). Evidence for a pathogenic role of nitric oxide in inflammation-induced osteoporosis. *Journal of Bone and Mineral Research*.

[14] Alnaeeli M, Teng YTA (2009). Dendritic cells: a new player in osteoimmunology. *Current Molecular Medicine*.

[15] Bar-Shavit Z (2007). The osteoclast: a multinucleated, hematopoietic-origin, bone-resorbing osteoimmune cell. *Journal of Cellular Biochemistry*.

[16] David JP (2007). Osteoimmunology: a view from the bone. *Advances in Immunology*.

[17] Barnabe C, Hanley DA (2009). Effect of tumor necrosis factor alpha inhibition on bone density and turnover markers in patients with rheumatoid arthritis and spondyloarthropathy. *Seminars in Arthritis and Rheumatism*.

[18] Di Virgilio F (2007). Liaisons dangereuses: P2X7 and the inflammasome. *Trends in Pharmacological Sciences*.

[19] Trautmann A (2009). Extracellular ATP in the immune system: more than just a ‘danger signal’. *Science Signaling*.

[20] La Sala A, Ferrari D, Di Virgilio F, Idzko M, Norgauer J, Girolomoni G (2003). Alerting and tuning the immune response by extracellular nucleotides. *Journal of Leukocyte Biology*.

[21] Wesselius A, Bours MJL, Agrawal A (2011). Role of purinergic receptor polymorphisms in human bone. *Frontiers in Bioscience*.

[22] Pellegatti P, Falzoni S, Pinton P, Rizzuto R, Di Virgilio F (2005). A novel recombinant plasma membrane-targeted luciferase reveals a new pathway for ATP secretion. *Molecular Biology of the Cell*.

[23] Pellegatti P, Raffaghello L, Bianchi G, Piccardi F, Pistoia V, Di Virgilio F (2008). Increased level of extracellular ATP at tumor sites: in vivo imaging with plasma membrane luciferase. *PLoS ONE*.

[24] Ferrari D, Pizzirani C, Adinolfi E (2006). The P2X7 receptor: a key player in IL-1 processing and release. *Journal of Immunology*.

[25] Ferrari D, Chiozzi P, Falzoni S (1997). Extracellular ATP triggers IL-1*β* release by activating the purinergic P2Z receptor of human macrophages. *Journal of Immunology*.

[26] Pelegrin P, Barroso-Gutierrez C, Surprenant A (2008). P2X7 receptor differentially couples to distinct release pathways for IL-1*β* in mouse macrophage. *Journal of Immunology*.

[27] Perregaux D, Gabel CA (1994). Interleukin-1*β* maturation and release in response to ATP and nigericin. Evidence that potassium depletion mediated by these agents is a necessary and common feature of their activity. *Journal of Biological Chemistry*.

[28] Labasi JM, Petrushova N, Donovan C (2002). Absence of the P2X7 receptor alters leukocyte function and attenuates an inflammatory response. *Journal of Immunology*.

[29] Ichinose M (1995). Modulation of phagocytosis by P2-purinergic receptors in mouse peritoneal macrophages. *Japanese Journal of Physiology*.

[30] Hanley PJ, Kronlage M, Kirschning C (2012). Transient P2X7 receptor activation triggers macrophage death independent of Toll-like receptors 2 and 4, caspase-1, and pannexin-1 proteins. *Journal of Biological Chemistry*.

[31] Lemaire I, Falzoni S, Zhang B, Pellegatti P, Di Virgilio F (2011). The P2X7 receptor and pannexin-1 are both required for the promotion of multinucleated macrophages by the inflammatory cytokine GM-CSF. *Journal of Immunology*.

[32] Pellegatti P, Falzoni S, Donvito G, Lemaire I, Di Virgilio F (2011). P2X7 receptor drives osteoclast fusion by increasing the extracellular adenosine concentration. *FASEB Journal*.

[33] Lopez-Castejon G, Theaker J, Pelegrin P, Clifton AD, Braddock M, Surprenant A (2010). P2X7 receptor-mediated release of cathepsins from macrophages is a cytokine-independent mechanism potentially involved in joint diseases. *Journal of Immunology*.

[34] Guerra AN, Fisette PL, Pfeiffer ZA (2003). Purinergic receptor regulation of LPS-induced signaling and pathophysiology. *Journal of Endotoxin Research*.

[35] Sluyter R, Wiley JS (2002). Extracellular adenosine 5′-triphosphate induces a loss of CD23 from human dendritic cells via activation of P2X7 receptors. *International Immunology*.

[36] Ferrari D, La Sala A, Chiozzi P (2000). The P2 purinergic receptors of human dendritic cells: identification and coupling to cytokine release. *FASEB Journal*.

[37] Sluyter R, Wiley JS (2002). Extracellular adenosine 5′-triphosphate induces a loss of CD23 from human dendritic cells via activation of P2X7 receptors. *International Immunology*.

[38] Ferrari D, La Sala A, Chiozzi P (2000). The P2 purinergic receptors of human dendritic cells: identification and coupling to cytokine release. *FASEB Journal*.

[39] Labasi JM, Petrushova N, Donovan C (2002). Absence of the P2X7 receptor alters leukocyte function and attenuates an inflammatory response. *Journal of Immunology*.

[40] Budagian V, Bulanova E, Brovko L (2003). Signaling through P2X7 receptor in human T cells involves p56lck MAP kinases, and transcription factors AP-1 and NF-*κ*B. *Journal of Biological Chemistry*.

[41] Bours MJL, Swennen ELR, Di Virgilio F, Cronstein BN, Dagnelie PC (2006). Adenosine 5′-triphosphate and adenosine as endogenous signaling molecules in immunity and inflammation. *Pharmacology and Therapeutics*.

[42] Buckley KA, Golding SL, Rice JM, Dillon JP, Gallagher JA (2003). Release and interconversion of P2 receptor agonists by human osteoblast-like cells. *FASEB Journal*.

[43] Grol MW, Zelner I, Dixon SJ (2012). P2X7-mediated calcium influx triggers a sustained, PI3K-dependent increase in metabolic acid production by osteoblast-like cells. *American Journal of Physiology—Endocrinology and Metabolism*.

[44] Liu D, Genetos DC, Shao Y (2008). Activation of extracellular-signal regulated kinase (ERK1/2) by fluid shear is Ca^2+^- and ATP-dependent in MC3T3-E1 osteoblasts. *Bone*.

[45] Matsuo K, Ray N (2004). Osteoclasts, mononuclear phagocytes, and c-Fos: new insight into osteoimmunology. *Keio Journal of Medicine*.

[46] Okumura H, Shiba D, Kubo T, Yokoyama T (2008). P2X7 receptor as sensitive flow sensor for ERK activation in osteoblasts. *Biochemical and Biophysical Research Communications*.

[47] Panupinthu N, Zhao L, Possmayer F, Ke HZ, Sims SM, Dixon SJ (2007). P2X7 nucleotide receptors mediate blebbing in osteoblasts through a pathway involving lysophosphatidic acid. *Journal of Biological Chemistry*.

[48] Gartland A, Hipskind RA, Gallagher JA, Bowler WB (2001). Expression of a P2X7 receptor by a subpopulation of human osteoblasts. *Journal of Bone and Mineral Research*.

[49] Panupinthu N, Rogers JT, Zhao L (2008). P2X7 receptors on osteoblasts couple to production of lysophosphatidic acid: a signaling axis promoting osteogenesis. *Journal of Cell Biology*.

[50] Orriss IR, Key ML, Brandao-Burch A (2012). The regulation of osteoblast function and bone mineralisation by extracellular nucleotides: the role of p2x receptors. *Bone*.

[51] Li J, Liu D, Ke HZ, Duncan RL, Turner CH (2005). The P2X7 nucleotide receptor mediates skeletal mechanotransduction. *Journal of Biological Chemistry*.

[52] Brandao-Burch A, Key ML, Patel JJ (2012). The P2X7 receptor is an important regulator of extracellular ATP levels. *Frontiers in Endocrinology*.

[53] Orriss IR, Knight GE, Utting JC, Taylor SEB, Burnstock G, Arnett TR (2004). Hransient exposure to hypoxia stimulates ATP release from osteoblasts. *Journal of Cellular Physiology*.

[54] Genetos DC, Karin NJ, Geist DJ, Donahue HJ, Duncan RL (2011). Purinergic signaling is required for fluid shear stress-induced NF-*κ*B translocation in osteoblasts. *Experimental Cell Research*.

[55] Gabel CA (2007). P2 purinergic receptor modulation of cytokine production. *Purinergic Signalling*.

[56] Penolazzi L, Bianchini E, Lambertini E (2005). N-Arylpiperazine modified analogues of the P2X7 receptor KN-62 antagonist are potent inducers of apoptosis of human primary osteoclasts. *Journal of Biomedical Science*.

[57] Korcok J, Raimundo LN, Ke HZ, Sims SM, Dixon SJ (2004). Extracellular nucleotides act through P2X7 receptors to activate NF-*κ*B in osteoclasts. *Journal of Bone and Mineral Research*.

[58] Jørgensen NR (2005). Short-range intercellular calcium signaling in bone. *APMIS, Supplement*.

[59] Brandao-Burch A, Burnstock G, Arnett T, Orriss I (2011). The P2X7 receptor is an important regulator of extracellular ATP levels. *Frontiers in Endocrinology*.

[60] Steinberg TH, Hiken JF (2007). P2 receptors in macrophage fusion and osteoclast formation. *Purinergic Signalling*.

[61] Gartland A, Buckley KA, Hipskind RA (2003). Multinucleated osteoclast formation in vivo and in vitro by P2X 7 receptor-deficient mice. *Critical Reviews in Eukaryotic Gene Expression*.

[62] Gartland A, Buckley KA, Hipskind RA, Bowler WB, Gallagher JA (2003). P2 receptors in bone—modulation of osteoclast formation and activity via P2X7 activation. *Critical Reviews in Eukaryotic Gene Expression*.

[63] Collo G, Neidhart S, Kawashima E, Kosco-Vilbois M, North RA, Buell G (1997). Tissue distribution of the P2X7 receptor. *Neuropharmacology*.

[64] Burnstock G, Knight GE (2004). Cellular distribution and functions of P2 receptor subtypes in different systems. *International Review of Cytology*.

[65] Gu BJ, Zhang WY, Bendall LJ, Chessell IP, Buell GN, Wiley JS (2000). Expression of P2X7 purinoceptors on human lymphocytes and monocytes: evidence for nonfunctional P2X7 receptors. *American Journal of Physiology—Cell Physiology*.

[66] Mariathasan S, Hewton K, Monack DM (2004). Differential activation of the inflammasome by caspase-1 adaptors ASC and Ipaf. *Nature*.

[67] Kahlenberg JM, Dubyak GR (2004). Mechanisms of caspase-1 activation by P2X7 receptor-mediated K+ release. *American Journal of Physiology—Cell Physiology*.

[68] Kahlenberg JM, Lundberg KC, Kertesy SB, Qu Y, Dubyak GR (2005). Potentiation of caspase-1 activation by the P2X7 receptor is dependent on TLR signals and requires NF-*κ*B-driven protein synthesis. *Journal of Immunology*.

[69] Dubyak GR (2012). P2X7 receptor regulation of non-classical secretion from immune effector cells. *Cellular Microbiology*.

[70] Pelegrin P, Surprenant A (2006). Pannexin-1 mediates large pore formation and interleukin-1*β* release by the ATP-gated P2X7 receptor. *EMBO Journal*.

[71] Tonetti M, Sturla L, Giovine M, Benatti U, De Flora A (1995). Extracellular ATP enhances mRNA levels of nitric oxide synthase and TNF-*α* in lipopolysaccharide-treated raw 264.7 murine macrophages. *Biochemical and Biophysical Research Communications*.

[72] Denlinger LC, Angelini G, Schell K (2005). Detection of human P2X7 nucleotide receptor polymorphisms by a novel monocyte pore assay predictive of alterations in lipopolysaccharide-induced cytokine production. *Journal of Immunology*.

[73] Grol MW, Panupinthu N, Korcok J, Sims SM, Dixon SJ (2009). Expression, signaling, and function of P2X7 receptors in bone. *Purinergic Signalling*.

[74] Henriksen Z, Hiken JF, Steinberg TH, Jørgensen NR (2006). The predominant mechanism of intercellular calcium wave propagation changes during long-term culture of human osteoblast-like cells. *Cell Calcium*.

[75] Jørgensen NR, Henriksen Z, Sørensen OH, Eriksen EF, Civitelli R, Steinberg TH (2002). Intercellular calcium signaling occurs between human osteoblasts and osteoclasts and requires activation of osteoclast P2X7 receptors. *Journal of Biological Chemistry*.

[76] Solgaard M, Jørgensen NR (2005). P2 purinergic receptors: their regulation of bone metabolism and their therapeutic potential. *Ugeskrift for Laeger*.

[77] Jørgensen NR, Geist ST, Civitelli R, Steinberg TH (1997). ATP- and gap junction-dependent intercellular calcium signaling in osteoblastic cells. *Journal of Cell Biology*.

[78] Jørgensen NR, Henriksen Z, Brot C (2000). Human osteoblastic cells propagate intercellular calcium signals by two different mechanisms. *Journal of Bone and Mineral Research*.

[79] Jørgensen NR, Teilmann SC, Henriksen Z, Civitelli R, Sørensen OH, Steinberg TH (2003). Activation of L-type calcium channels is required for gap junction-mediated intercellular calcium signaling in osteoblastic cells. *Journal of Biological Chemistry*.

[80] Kringelbach T, Novak I, Bonewald L (2012). UTP and mechanical stimulation induce ATP release from osteocytes. *Bone*.

[81] Gallagher JA, Buckley KA (2002). Expression and function of P2 receptors in bone. *Journal of Musculoskeletal Neuronal Interactions*.

[82] Gallagher JA (2004). ATP P2 receptors and regulation of bone effector cells. *Journal of Musculoskeletal Neuronal Interactions*.

[83] Orriss IR, Key ML, Brandao-Burch A (2012). The regulation of osteoblast function and bone mineralisation by extracellular nucleotides: the role of p2x receptors. *Bone*.

[84] Genetos DC, Geist DJ, Liu D, Donahue HJ, Duncan RL (2005). Fluid shear-induced ATP secretion mediates prostaglandin release in MC3T3-E1 osteoblasts. *Journal of Bone and Mineral Research*.

[85] Gartland A, Buckley KA, Hipskind RA (2003). Multinucleated osteoclast formation in vivo and in vitro by P2X 7 receptor-deficient mice. *Critical Reviews in Eukaryotic Gene Expression*.

[86] Liu D, Genetos DC, Shao Y (2008). Activation of extracellular-signal regulated kinase (ERK1/2) by fluid shear is Ca^2+^- and ATP-dependent in MC3T3-E1 osteoblasts. *Bone*.

[87] Panupinthu N, Zhao L, Possmayer F, Ke HZ, Sims SM, Dixon SJ (2007). P2X7 nucleotide receptors mediate blebbing in osteoblasts through a pathway involving lysophosphatidic acid. *Journal of Biological Chemistry*.

[88] Kringelbach T, Schwarz P, Novak I (2008). P2 receptors are functionally expressed in MLO-Y4 osteocytes. *Purinergic Signalling*.

[89] Li J, Liu D, Ke HZ, Duncan RL, Turner CH (2005). The P2X7 nucleotide receptor mediates skeletal mechanotransduction. *Journal of Biological Chemistry*.

[90] Kringelbach T, Novak I, Schwarz P, Jørgensen NR (2013). Nucleotide and mechanically induced ATP release pathways in osteocytes. *Bone Abstracts*.

[91] Brandao-Burch A, Key ML, Patel JJ (2012). The P2X7 receptor is an important regulator of extracellular ATP levels. *Frontiers in Endocrinology*.

[92] Takayanagi H (2005). Mechanistic insight into osteoclast differentiation in osteoimmunology. *Journal of Molecular Medicine*.

[93] Arbor A (2009). Inhibition of osteoblast functions by IKK/NF-*κ*B in osteoporosis. *Nature Medicine*.

[94] Tak PP, Firestein GS (2001). NF-*κ*B: a key role in inflammatory diseases. *Journal of Clinical Investigation*.

[95] Marcus R, Feldman D, Nelson D, Rosen C (2009). *Fundamentals of Osteoporosis*.

[96] Grol MW, Zelner I, Jeffrey Dixon S (2012). P2X7-mediated calcium influx triggers a sustained, PI3K-dependent increase in metabolic acid production by osteoblast-like cells. *American Journal of Physiology—Endocrinology and Metabolism*.

[97] Ke HZ, Qi H, Weidema AF (2003). Deletion of the P2X7 nucleotide receptor reveals its regulatory roles in bone formation and resorption. *Molecular Endocrinology*.

[98] Gu BJ, Wiley JS (2006). Rapid ATP-induced release of matrix metalloproteinase 9 is mediated by the P2X7 receptor. *Blood*.

[99] Jelassi B, Chantme A, Alcaraz-Pérez F (2011). P2X 7 receptor activation enhances SK3 channels- and cystein cathepsin-dependent cancer cells invasiveness. *Oncogene*.

[100] Solle M, Labasi J, Perregaux DG (2001). Altered cytokine production in mice lacking P2X7 receptors. *Journal of Biological Chemistry*.

[101] Xu X, Boumechache M (2012). Splice-variants of the P2X7 receptor reveal differential agonist-dependence and functional coupling with pannexin-1. *Journal of Cell Science*.

[102] Nicke A, Kuan YH, Masin M (2009). A functional P2X7 splice variant with an alternative transmembrane domain 1 escapes gene inactivation in P2X7 knock-out mice. *Journal of Biological Chemistry*.

[103] Cheewatrakoolpong B, Gilchrest H, Anthes JC, Greenfeder S (2005). Identification and characterization of splice variants of the human P2X7 ATP channel. *Biochemical and Biophysical Research Communications*.

[104] Masin M, Young C, Lim K (2012). Expression, assembly and function of novel C-terminal truncated variants of the mouse P2X7 receptor: re-evaluation of P2X7 knockouts. *British Journal of Pharmacology*.

[105] Syberg S, Petersen S, Beck Jensen JE (2012). Genetic background strongly influences the bone phenotype of P2X7 receptor knockout mice. *Journal of Osteoporosis 2012*.

[106] Syberg S, Schwarz P, Petersen S (2012). Association between P2X7 receptor polymorphisms and bone status in mice. *JournAl of Osteoporosis*.

[107] Orriss I, Syberg S, Wang N (2011). Bone phenotypes of P2 receptor knockout mice. *Frontiers in Bioscience*.

[108] Ohlendorff SD, Tofteng CL, Jensen JEB (2007). Single nucleotide polymorphisms in the P2X7 gene are associated to fracture risk and to effect of estrogen treatment. *Pharmacogenetics and Genomics*.

[109] Jørgensen NR, Husted LB, Skarratt KK (2012). Single-nucleotide polymorphisms in the P2X7 receptor gene are associated with post-menopausal bone loss and vertebral fractures. *European Journal of Human Genetics*.

[110] Gartland A, Skarratt KK, Hocking LJ (2012). Polymorphisms in the P2X7 receptor gene are associated with low lumbar spine bone mineral density and accelerated bone loss in post-menopausal women. *European Journal of Human Genetics*.

[111] Wesselius A, Bours MJL, Agrawal A (2011). Role of purinergic receptor polymorphisms in human bone. *Frontiers in Bioscience*.

[112] Wesselius A, Bours MJL, Henriksen Z (2013). Association of P2X7 receptor polymorphisms with bone mineral density and osteoporosis risk in a cohort of Dutch fracture patients. *Osteoporosis International*.

[113] Portales-Cervantes L, Niño-Moreno P, Doníz-Padilla L (2010). Expression and function of the P2X7 purinergic receptor in patients with systemic lupus erythematosus and rheumatoid arthritis. *Human Immunology*.

[114] Pelegrin P (2008). Targeting interleukin-1 signaling in chronic inflammation: focus on P2X(7) receptor and Pannexin-1. *Drug News & Perspectives*.

[115] Roger S, Pelegrin P (2011). P2X7 receptor antagonism in the treatment of cancers. *Expert Opinion on Investigational Drugs*.

[116] Takenouchi T, Sekiyama K, Sekigawa A (2010). P2X7 Receptor signaling pathway as a therapeutic target for neurodegenerative diseases. *Archivum Immunologiae et Therapiae Experimentalis*.

[117] Arulkumaran N, Unwin RJ, Tam FWK (2011). A potential therapeutic role for P2X7 receptor (P2X7R) antagonists in the treatment of inflammatory diseases. *Expert Opinion on Investigational Drugs*.

[118] Baraldi PG, Di Virgilio F, Romagnoli R (2004). Agonists and antagonists acting at P2X7 receptor. *Current Topics in Medicinal Chemistry*.

[119] Romagnoli R, Baraldi PG, Cruz-Lopez O (2008). The P2X7 receptor as a therapeutic target. *Expert Opinion on Therapeutic Targets*.

[120] Friedle SA, Curet MA, Watters JJ (2010). Recent patents on novel p2x7 receptor antagonists and their potential for reducing central nervous system inflammation. *Recent Patents on CNS Drug Discovery*.

[121] Keystone EC, Wang MM, Layton M, Hollis S, McInnes IB (2012). Clinical evaluation of the efficacy of the P2X7 purinergic receptor antagonist AZD9056 on the signs and symptoms of rheumatoid arthritis in patients with active disease despite treatment with methotrexate or sulphasalazine. *Annals of the Rheumatic Diseases*.

[122] Stock TC, Bloom BJ, Wei N (2012). Efficacy and safety of CE-224,535, an antagonist of P2X7 receptor, in treatment of patients with rheumatoid arthritis inadequately controlled by methotrexate. *Journal of Rheumatology*.

[123] Cruwys S, Midha A, Braddock M Antagonism of the P2X7 receptor Attenuates Joint Destruction in a Model of Arthrites.

